# Supplementary material for: Disease-associated mutations in WDR34 lead to diverse impacts on the assembly and function of dynein-2
Source: J Cell Sci. 2022 Nov 7;136(5):jcs260073. doi: 10.1242/jcs.260073 (PMC9687537; doi:10.1242/jcs.260073)
Supplement: Supplementary information [file joces-136-260073-s1.pdf]

Supplementary Figure S1

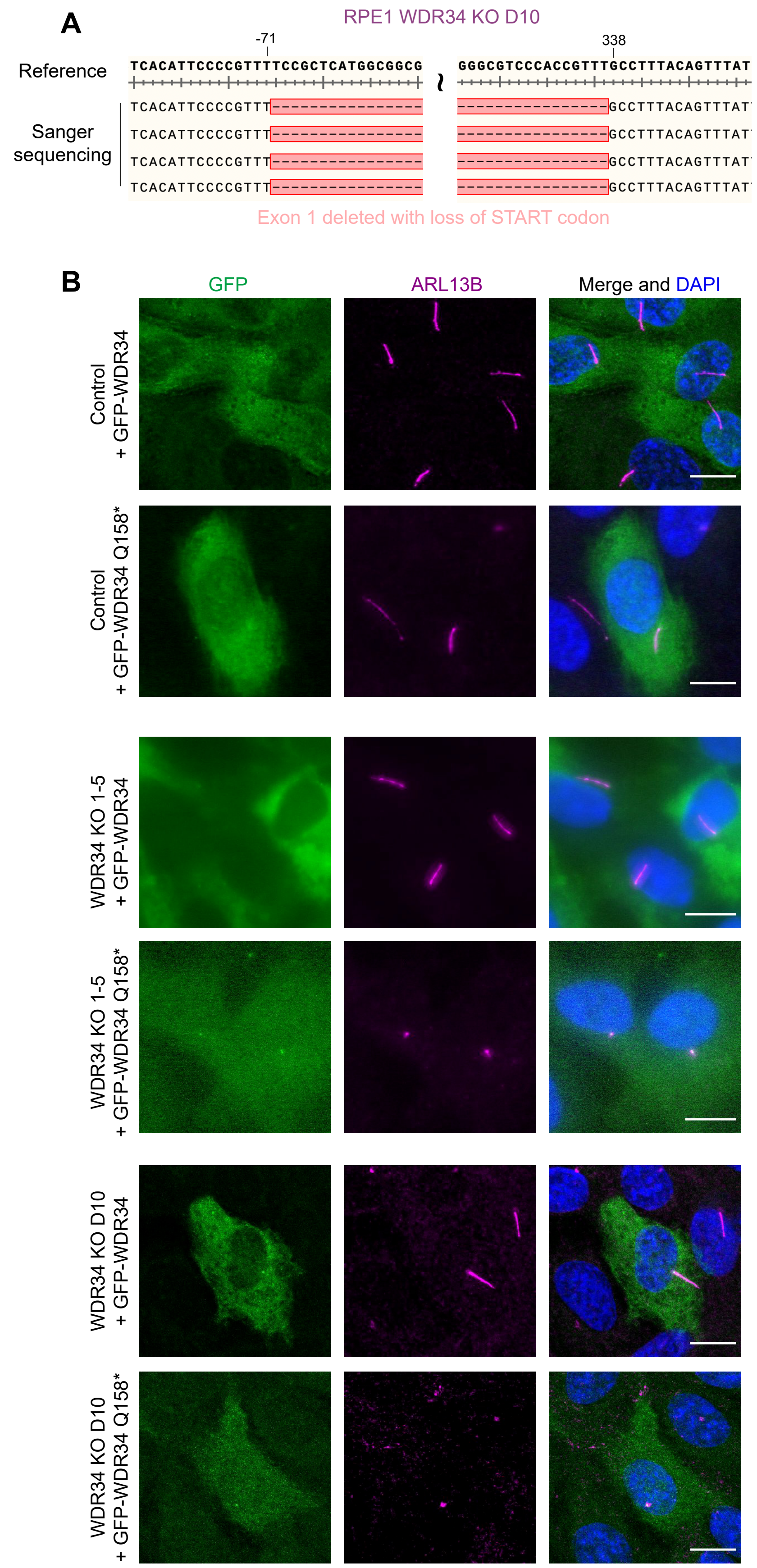

Fig. S1. Generation of WDR34 KO RPE-1 cell line and characterization of effect of WDR34 p.Q158* on cilia formation. (A) Alignments of sequence of WDR34 KO cell line D10 determined by Sanger sequencing of genomic DNA PCR product with reference sequence. PAM is highlighted with red box. Large deletion of 410 bp is observed. (B) Translation product of WDR34 gene of WDR34 KO vs Control cell line. Red arrow indicates complete loss of exon 1 (light-chain binding sites) along with loss of START codon. (C) GFP-WDR34-p.Q158* and GFP-WDR34 (WT) stably expressed in either control, WDR34 KO D10 and WDR34 KO 1-5 background were serum-starved for 24 h to induce ciliogenesis and stained with Arl13b and acetylated tubulin antibodies. Scale bar = 10 μm.

Journal of Cell Science • Supplementary information

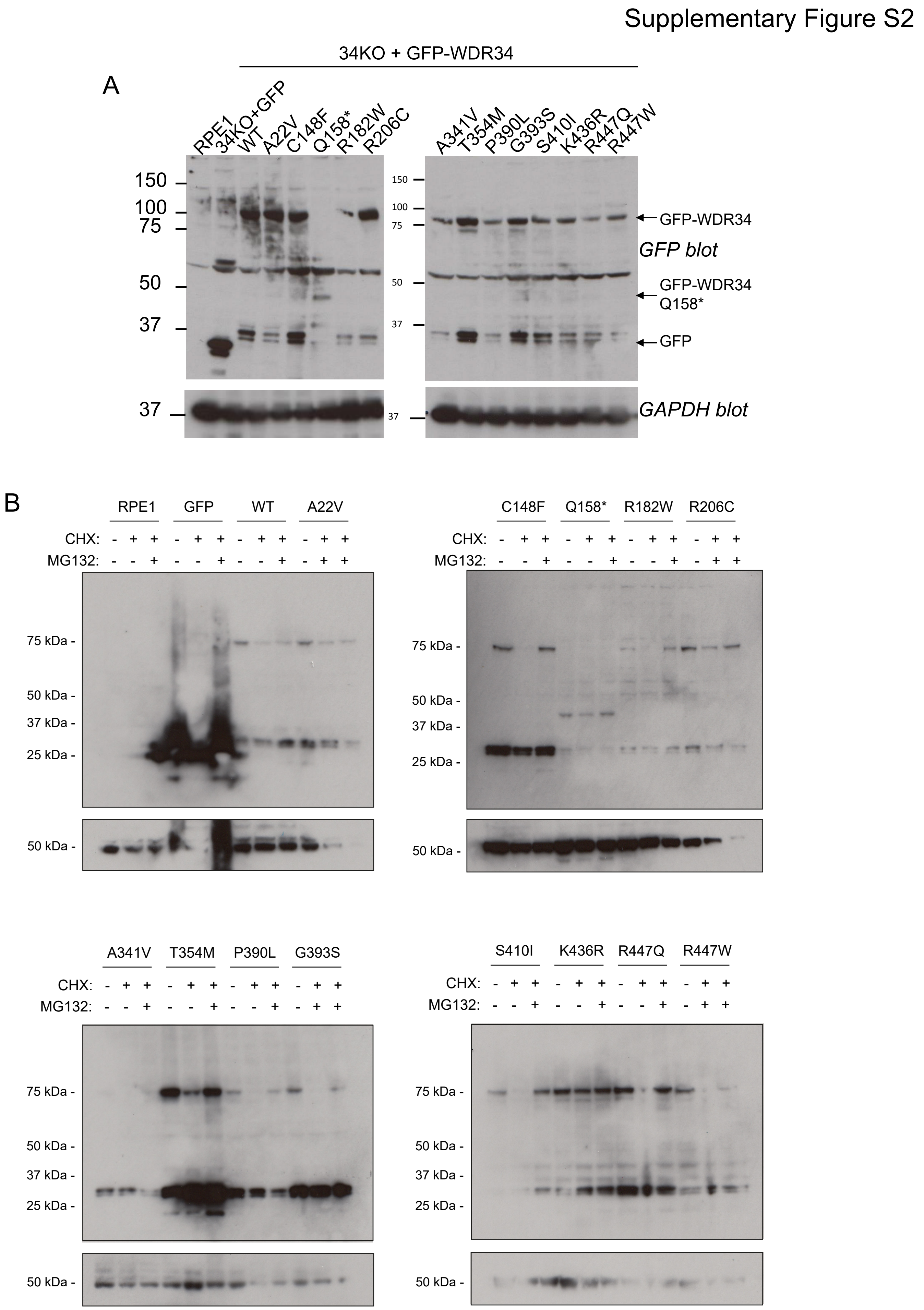

Fig. S2. (A) Expression of GFP-WDR34 mutants was confirmed by immunoblotting using an anti-GFP antibody with GAPDH as a loading control. Molecular weights are shown in kDa. Note that all lanes show a non-specific band at ~60 kDa. (B) Full gels from the experiment shown in Figure 2C including tubulin as an additional control.

Journal of Cell Science • Supplementary information

Supplementary Figure S3

	**A22V**	**C148F**	**Q158***	**R182W**
**Human**	GVAALATVGVA	QQMVSCLYTLG	GYPPAQAQGLH	ACAYGRLDHGD
**Chimpanzee**	GVAALATVGVA	QQMVSCLYTLG	GYPPAQAQGLH	ACAYGRLDHGD
**Mouse**	GAAALAAGGAG	QQTVSCLHTLV	VYPLAQGQGLH	ACAYGRLDDGD
**Cat**	GAEALATGGAA	QPTVTCLHTLG	GHPPAQGQGLH	ACAYGRLDDGD
**Dog**	GAEALATGGAA	QPTVTCLHTLG	GYPPAQGQGLH	ACAYGRLDDGD
**Chicken**	--SA--FTACP	NRTVLCLHTLS	SYPEAQDQHLQ	ACSYGRLNDGD
**Zebrafish**	-----------	NESVSCMYRLQ	QHVDAQEKSLQ	ACGFGRVDDGD

	**R206C**	**A341V**	**T354M**	**P390L G393S**
**Human**	DRRDLRPQQPS	GATAVAFSSFD	LFILGTEGGFP	QFTFSPHGGPIYSV
**Chimpanzee**	DRRDLHPQQPS	GATAVAFSSFD	LFILGTEGGFP	QFTFSPHGGPIYSV
**Mouse**	DRQGLNPQQPS	GVTSVAFSSFD	LFVLGTEGGFP	QFTFSPHGGPVYSV
**Cat**	DRRGLNPQQPS	GATAVAFSGFD	LFVLGTEGGFP	RFTFSPHGGPIYSV
**Dog**	DRRGLNPQQPS	GATAVAFSSFD	LFVLGTEGGFP	QLIFSPHGGPIYSV
**Chicken**	DRRRLDPQRPD	GVTSLSFSHFD	VFIVGVEGGYS	ELAFSPHSGPLYSV
**Zebrafish**	DRQNLNPKRPD	GVTAVALSPWD	TFLVGSEGGLV	QFSFSPRGGPIHSV

	**S410I**	**K436R**	**R447Q/W**
**Human**	RNLFLSAGTDG	LQLSLKYLFAV	RWSPVRPLVFA
**Chimpanzee**	RNLFLSAGTDG	LQLSLKYLFAV	RWSPVRPLVFA
**Mouse**	RNLFLSAGTDG	LQLSHKYLFAV	RWSPVRPLVFA
**Cat**	RNLFLSAGTDG	LQLSHKYLFAV	RWSPVRPLVFA
**Dog**	RNLFLSAGTDG	LQLSHKYLFAV	RWSPVRPLVFA
**Chicken**	RNLFLSCGTDG	LQLSTKYLFCV	RWSPVRPLVFA
**Zebrafish**	RNLFVSVGTDG	LRVSDSYVFGV	RWSPTRPLVFA

Fig. S3. Multiple sequence alignment of WDR34 showing the location of clinical mutations. Well-conserved residues are highlighted in blue. p.R206C is poorly conserved between species (yellow highlight). Alignments were produced using the multiple sequence alignment tool T-Coffee.

Journal of Cell Science • Supplementary information

Supplementary Figure S4

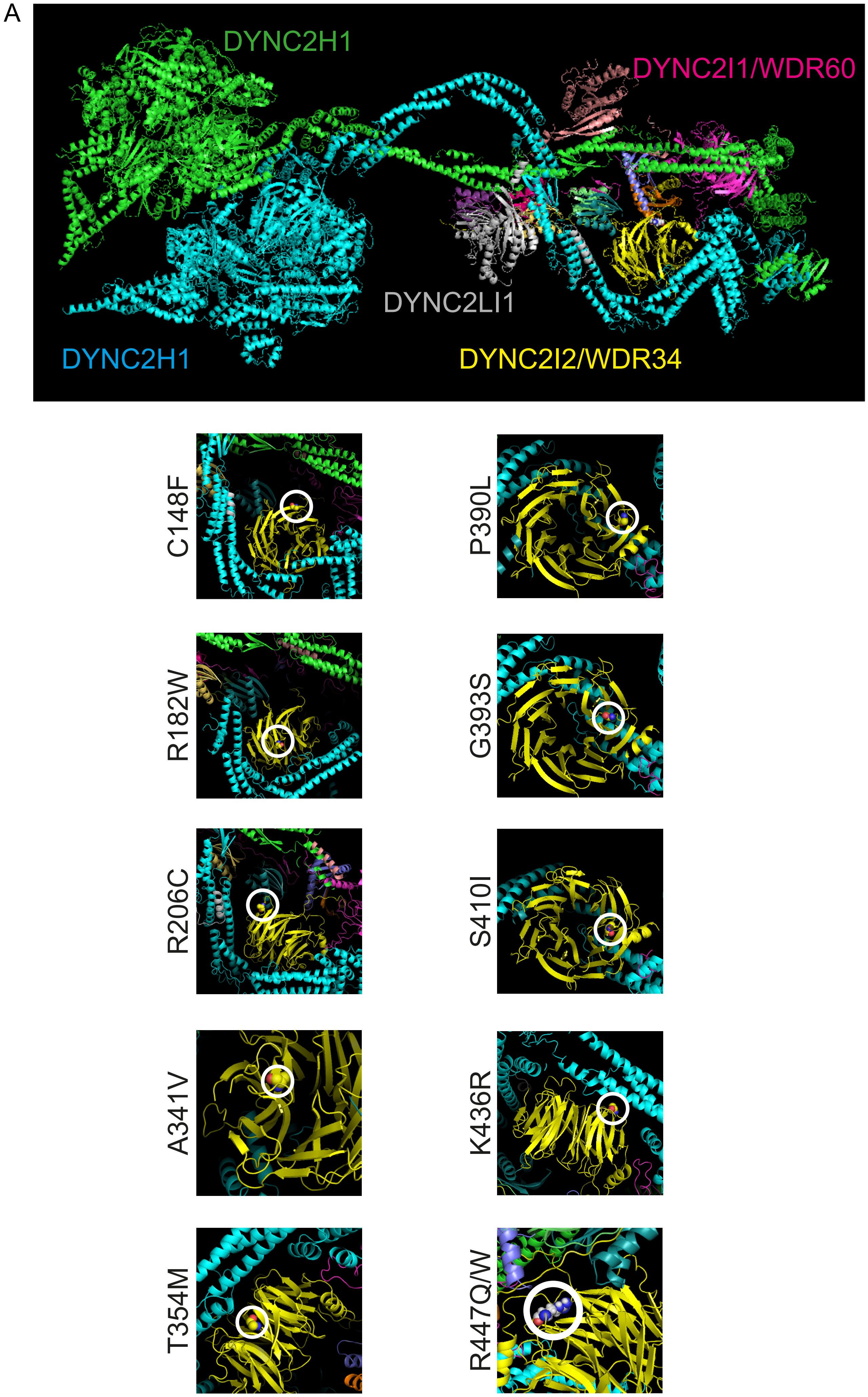

Journal of Cell Science • Supplementary information

Fig. S4. (A) Structural representation of the mutations investigated in this study in the context of the intact inactive state of the dynein-2 complex (Toropova et al., 2019, PDB 6SC2 (dynein-2, docked into subtomogram average of the anterograde IFT-B train (Jordan et al., 2018, EMDB-4303)). (B) The location of each mutation is shown and highlighted by a white circle; the original residues are shown in each case, not that arising from mutation. Figures were prepared using Pymol.

Journal of Cell Science • Supplementary information

Supplementary Figure S5

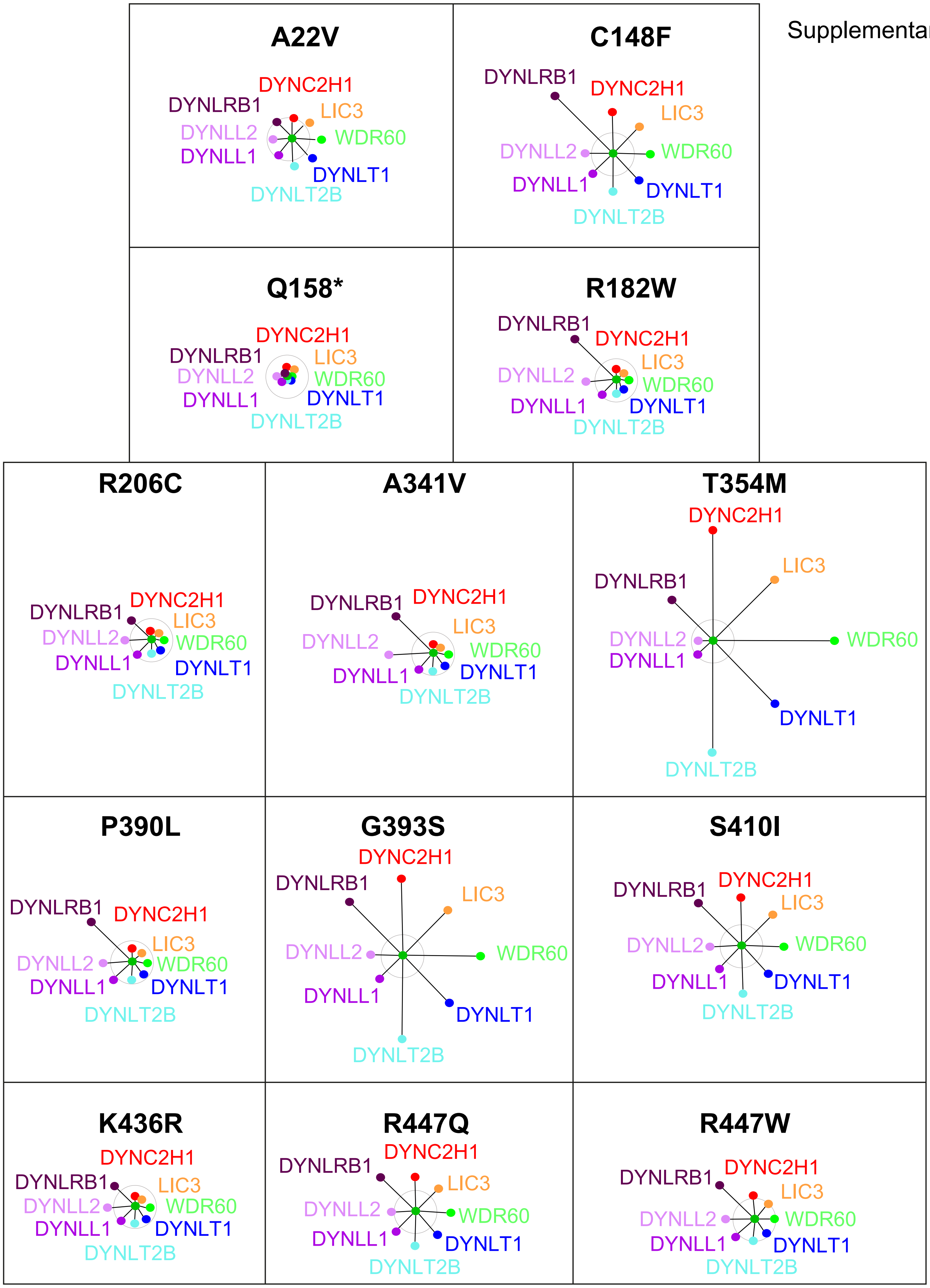

Fig. S5. Schematic representations of proteomic data. In each case, the circle indicates a ratio of 1 between mutant and wild-type WDR34 (i.e. no impact). Those subunits lying within the circle are more tightly associated with GFP-WDR34, those outside are less tightly associated. Distances indicate the inverse of the ratios shown in Figure 1.

Table S1. Clinical impact of disease-causing variants in *WDR34*. Table provides a brief description of the clinical impact of each mutation along with some contextual comments and a reference to the original descriptions.

Mutation	Clinical impact	Comments	Reference
c.65C>T (p.A22V)	Skeletal defects	Homozygous missense variant identified with homozygous *DYNC2H1* and compound heterozygous variant in *IFT140* c.4058C>G;	Schmidts et al., 2013
c.443G>T (p.C148F)	Polyhydramnios and skeletal defects with protruding abdomen, hepatic ptosis.	Compound heterozygous with c.1372+1G>A	Schmidts et al., 2013
c.472C>T (p.Q158*)	Skeletal defects with rod-cone dystrophy, obesity, and speech and language delay.	Compound heterozygous missense with c.1307A>G (K436R)	Schmidts et al., 2013
c.544C>T (p.R182W)	Skeletal defects polydactyly, lung and pulmonary problems	Homozygous missense variant	You et al., 2017
c.616C>T (p.R206C)	Skeletal defects with severe respiratory distress, recurrent infections, home ventilation.	Homozygous missense variant	Schmidts et al., 2013
c.1169C>T (p.P390L)	Skeletal defects with bilateral nephrocalcinosis but no cysts.	Homozygous missense variant identified as heterozygous with *WDR19* c.2720C>T	Schmidts et al., 2013
c.1022C>T (p.A341V)	Skeletal defects and, in one case, hypotrophic lungs	Homozygous missense variant	Huber et al., 2013
c.1061C>T (p.T354M)	Skeletal defects	Homozygous missense variant	Huber et al., 2013
c.1177G>A (p.G393S)	Skeletal defects	Missense variant also identified as Compound heterozygous with c.1541_1542delCA (p.T514Afs*11, is not included in this study).	Schmidts et al., 2013

Journal of Cell Science • Supplementary information

c.1229G>T (p.S410I)	Skeletal defects and electroretinogram at lower limit of normal	Homozygous missense variant	Schmidts et al., 2013
c.1339C>T (p.R447W)	Skeletal defects polyhydramnios, elevated csf spaces, foot malformation, umbilical hernia, respiratory insufficiency, elevated cerebrospinal fluid spaces, foot malformation, umbilical hernia. Also identified by Huber et al 2013 in a case with skeletal defects and polydactyly.	Homozygous missense variant	Schmidts et al., 2013 and Huber et al., 2013.
c.1340G>A (p.R447Q)	Skeletal defects	Compound heterozygous missense variant with c.982-2T>G	Huber et al., 2013

Table S2. Predictions of the functional impact of disease-causing variants in WDR34. Scores from PROVEAN and PolyPhen2 (PPH2) are shown; we use the prediction terminology from each algorithm which are colour coded according to impact with neutral/benign in blue, possibly deleterious in yellow, and deleterious/probably deleterious in red. FPR indicates the False Positive Rate (1 – specificity at the indicated probability), TPR the True positive rate (sensitivity at the indicated probability). SIFT (Sorting Intolerant From Tolerant, (Ng and Henikoff, 2003)) and Grantham (Grantham, 1974) scores are also included.

Mutation	PPH2 HumDiv prediction	PPH2 probability	PPH2 FPR	PPH2 TPR	PPH2 HumVar prediction	PPH2 probability	PPH2 FPR	PPH2 TPR	PROVEAN score	PROVEAN prediction (cutoff - 2.5)	SIFT score	Grantham score
A22V	Probably damaging	0.969	0.045	0.77	Benign	0.304	0.234	0.861	-0.848	Neutral	0	64
C148F	Benign	0.251	0.118	0.911	Benign	0.145	0.29	0.895	-6.533	Deleterious	0.06	205
R182W	Probably damaging	0.998	0.0112	0.273	Possibly damaging	0.878	0.109	0.711	-2.362	Neutral	0.19	101
R206C	Possibly damaging	0.846	0.0675	0.834	Benign	0.042	0.382	0.932	-2.738	Deleterious	0.03	180
A341V	Benign	0.34	0.11	0.9	Benign	0.033	0.4	0.937	-2.137	Neutral	0.05	64
T354M	Probably damaging	0.995	0.0277	0.681	Possibly damaging	0.676	0.154	0.788	-6.658	Deleterious	0	81
P390L	Probably damaging	0.962	0.0478	0.779	Possibly damaging	0.89	0.106	0.704	-5.142	Deleterious	0.1	98
G393S	Probably damaging	0.999	0.00574	0.136	Probably damaging	0.983	0.0589	0.558	-3.375	Deleterious	0.16	56
S410I	Possibly damaging	0.946	0.0537	0.795	Possibly damaging	0.583	0.173	0.81	-0.782	Neutral	0.01	142
K436R	Benign	0	1	1	Benign	0.001	0.912	0.994	-3.439	Deleterious	0.46	26
R447Q	Probably damaging	1	0.00026	0.00018	Probably damaging	0.994	0.0403	0.463	-6.878	Deleterious	0.21	43
R447W	Probably damaging	1	0.00026	0.00018	Probably damaging	0.999	0.00759	0.0901	-6.154	Deleterious	0.01	101

Table S3. Abundance ratios of dynein-2 subunits found in association with GFP-WDR34-FL and GFP-WDR34-Gln158*. Data are shown from two independent experiments. The abundances were normalized to peptide counts for GFP to account for variation in expression level.

	Replicate 1				Replicate 2			
	Unique Peptides	Normalised abundances		Abundance ratio	Unique Peptides	Normalised abundances		Abundance ratio
NAME		GFP-WDR34-Gln158*	GFP-WDR34	GFP-WDR34-Gln158* / GFP-WDR34		GFP-WDR34-Gln158*	GFP-WDR34	GFP-WDR34-Gln158* / GFP-WDR34
DYNC2H1	163	17.4	12.6	1.4	149	27.2	14.5	1.9
WDR60	36	9.6	1.7	5.8	37	15.7	2.6	6
DYNLL1	2	2.0	1.0	1.9	2	3.1	1.5	2.1
DYNLRB1	4	3.1	0.6	5.0	3	2.5	0.6	4.4
DYNC2LI1	10	0.7	0.6	1.3	9	1.4	0.8	1.8
DYNLL2	1	0.3	0.1	2.0	2	0.4	0.3	1.7
DYNLT1	1	0.3	0.1	4.8	1	0.4	0.1	5.5
TCTEX1D2	6	1.1	0.2	5.6	4	1.8	0.3	5.7
