# Supplementary material for: Disease-associated mutations in WDR34 lead to diverse impacts on the assembly and function of dynein-2
Source: J Cell Sci. 2022 Nov 7;136(5):jcs260073. doi: 10.1242/jcs.260073 (PMC9687537; doi:10.1242/jcs.260073)
Supplement: Supplementary file 1 [file joces-136-260073_review_history.pdf]

Journal of Cell Science

Disease-associated mutations in WDR34 lead to diverse impacts on the assembly and function of dynein-2

Caroline Shak, Borhan Uddin, Laura Vuolo, Yohei Katoh, Tom Brown, Aakash G. Mukhopadhyay, Kate Heesom, Nicola Stevenson, Anthony John Roberts, Kazuhisa Nakayama and David J. Stephens
DOI: 10.1242/jcs.260073

Editor: Anne Straube

Review timeline

Original submission:	1 April 2022
Editorial decision:	16 May 2022
First revision received:	26 August 2022
Editorial decision:	26 September 2022
Second revision received:	10 October 2022
Accepted:	12 October 2022

Original submission

First decision letter

MS ID#: JOCES/2022/260073

MS TITLE: Clinical mutations in WDR34 lead to diverse impacts on the assembly and function of dynein-2

AUTHORS: Caroline Shak, Borhan Uddin, Laura Vuolo, Yohei Katoh, Tom Brown, Kate Heesom, Nicola Stevenson, Kazuhisa Nakayama, and David Stephens
ARTICLE TYPE: Research Article

We have now reached a decision on the above manuscript.

To see the reviewers' reports and a copy of this decision letter, please go to: https://submit-jcs.biologists.org and click on the 'Manuscripts with Decisions' queue in the Author Area. (Corresponding author only has access to reviews.)

As you will see, the reviewers raise a number of substantial criticisms that prevent me from accepting the paper at this stage. They suggest, however, that a revised version might prove acceptable, if you can address their concerns. If you think that you can deal satisfactorily with the criticisms on revision, I would be pleased to see a revised manuscript. We would then return it to the reviewers.

Please ensure that you clearly highlight all changes made in the revised manuscript. Please avoid using 'Tracked changes' in Word files as these are lost in PDF conversion.

I should be grateful if you would also provide a point-by-point response detailing how you have dealt with the points raised by the reviewers in the 'Response to Reviewers' box. Please attend to all of the reviewers' comments. If you do not agree with any of their criticisms or suggestions please explain clearly why this is so.

© 2022. Published by The Company of Biologists under the terms of the Creative Commons Attribution License (https://creativecommons.org/licenses/by/4.0/).

Reviewer 1

Advance summary and potential significance to field

Shak and colleagues use a cell culture model to characterize 13 disease-causing mutations in the dynein-2 subunit WDR34 (12 missense mutations and 1 nonsense mutation).

Using lentiviral transduction of RPE-1 cells in which WDR34 has been knocked out the authors generate transgenic cell lines that stably over-express GFP-tagged WDR34 mutants. These are compared with cells expressing GFP on its own and GFP-tagged wild-type (WT) WDR34.

Quantitative proteomic analysis after isolation of the GFP tag reports on dynein-2 holocomplex composition, and immunofluorescence-based assays are used to assess the efficiency of cilia formation, cilia length, transition zone integrity intraflagellar transport, and hedgehog signaling. Each of the 13 mutants differs from the WT control in at least one of the assays and no two mutants behave the same in all assays. Interestingly, the nonsense mutant has a more severe effect than loss of WDR34, presumably because the truncated WDR34 acts as a dominant-negative.

As the authors acknowledge in the discussion section, the transgenic over-expression approach comes with a significant caveat: since the expression level of WDR34 mutants is likely to be much higher than that of endogenous WDR34, it is questionable whether the cilia phenotype observed in this cell culture model accurately reflects what occurs in patient cells. Nevertheless, for most of these WDR34 mutations this is the first study that examines their impact on cilia in detail, and the results therefore make a valuable contribution to our understanding of ciliopathies caused by defects in the dynein-2 transport machinery.

Comments for the author

Main concern:
The majority of WDR34 mutants appear to be expressed at significantly lower levels than the WT control, which complicates the interpretation of the phenotypes. In cases where the mutant phenotype is different from WT, it is unclear to what extent this is due to differences in expression levels. Differences in expression levels among the mutants is another confounding factor. For example when discussing the effects of R447W and R447Q (lines 382 - 388), the authors state that “the differences between the Q and W at this position suggests some critical differences”. Judging by the immunoblot in Figure 1B, these “critical differences” may well come down to different expression levels. The authors should acknowledge the potential impact of variable protein levels. The authors should also provide an explanation for this variability in expression levels. Do some mutations prevent proper folding of WDR34, resulting in degradation of the mutant protein? One simple way to test this would be to inhibit proteasome activity, which in case of a destabilizing mutation would be predicted to increase mutant protein levels. The authors state that FACS was used in the generation of the cell lines but don’t mention single-cell sorting: are these cell lines clonal? If so, were expression levels similar among different clones, i.e. was it impossible to obtain mutant expression levels that are similar to the WT control?

Specific comments:
- Title: is “clinical mutations” a commonly used term? Maybe change to “disease-causing mutations” or "disease-associated mutations".
- Line 41: add “respectively”.
- Lines 45/46: “different spectrums of phenotypes” and “distinct profile of phenotypes” is repetitive.
- Figure 1B: the immunoblot needs to be of better quality so that relative expression levels can be properly assessed. The GAPDH signal is overexposed, which makes it difficult to judge loading. The GFP signal for WT GFP-WDR34 is also overexposed. The “RPE-1”, “34KO+GFP”, and “WT” samples should be included in both blots, not just in the one on the left.
- Figure 1C: A description of the proteomic analysis should be included in the materials and methods section. It would also be good to confirm at least some of these differences by immunoblot (for example DYNC2H1 levels in samples R206C vs T354M), but this is only a suggestion.
- Line 193: “Interestingly, apart from WDR34-K436R, interaction with DYNLRB1 was reduced by more than half in all WDR34 point mutants located in a WD domain suggesting defective assembly

© 2022. Published by The Company of Biologists under the terms of the Creative Commons Attribution License (https://creativecommons.org/licenses/by/4.0/).

of DYNLRB1 in dynein-2 may be a common phenotype in WDR34-related disorders". The relative abundance of DYNLRB1 in the control sample
"WT-WDR34" is also reduced by more than half, so I don't understand how the authors came to this conclusion.
- Figure 2A: why is the GFP signal in "34KO+GFP" so spotty?
- Lines 238 - 240: repetition
- Figure 4: Given the non-specific nuclear dots of the IFT140 staining, it would be helpful to add an arrow to the IFT140 images that points at the ciliary base.
- Line 250/Figure 4: "This complicates analysis and so we have not quantified these data but instead scored for the robust presence of IFT140 by eye." Do the authors mean that the unspecific signal prevents *automated* image analysis?
Scoring localization "by eye" is also a form of quantification, is it not?
- Line 259: "We next investigated the localization of the IFT-B protein, IFT88, as a well-characterized reporter of IFT defects." Why a reporter of IFT "defects"?
- Line 265: "WDR34 WT rescued IFT88 distribution from the cilium length." Why cilium "length"?
- Line 308: why "unfortunately"?
- Line 354: "Modelling of the mutations indicated that A341V leads to instability of the beta-propellor structure, with the mutant valine clashing with the first strand of the next propellor blade. Instead, T354 may form important hydrogen bonds important for maintaining structure stability, which are lost upon mutation into methionine." How was this modeling performed?
- Figure S3: Include the WT sample.
- Figure S4: It would be useful to move this summary table to the last main figure. Rather than just "yes" or "no", the extent of the effect could also be indicated (e.g. "+" vs "++").

Reviewer 2

Advance summary and potential significance to field

Ciliopathies are an expanding group of genetics disorders characterized by defects in the structure and/or function of cilia, small microtubule-based projections found on most mammalian cell types. There is a growing disconnect between genotype-phenotype correlations between ciliopathy patients in terms of expressivity and penetrance of clinical phenotypes even between individuals with mutations in the same gene, sometimes even same mutation. The reason for this pleiotropy is not well-understood but impinges on our clinical diagnosis and management of ciliopathy patients. Better ways of linking genotype to molecular and cellular phenotypes to inform on physiology are needed. Shak et al. attempt to do this here for the variants of the IFT dynein-2 intermediate chains DYNC2I2/WDR34 implicated in a spectrum of human skeletal dysplasias. They use a KO human ciliated epithelial line to over-express 13 disease-causing variants to look at molecular motor assembly by proteomics, as well as cilia structure (IFT trafficking by IF) and function (SMO localization by SAG stimulation). The narrative needs attention in terms of what this study is trying to do upfront and why it should target an audience like JCS as opposed to Human Molecular Genetics, for example, where these types of studies normally sit. Additional figure panels to help the audience understand the molecular asymmetry of dynein-2 with a schematic, where colors match your IP legends in figure 1C for example, possibly highlighting the subcomplexes you see partially assembling. It would also benefit from a systemic structural analysis of the mutations (from three papers) through a single predictive pipeline in terms of pathogenicity- (at a minimum PolyPhen, SIFT and Grantham scores, but there are fancier tools out there too…)- a table such that it is a ranked list of predicted severity, is this what you see in your cell assays? Finally, a model summarizing what you have found at the end would help bring together all these experimental strands which currently feel very frayed together again, what is the punchline of these analyses. This is important and timely work but some additional efforts to increase accessibility of data and messaging would help a JCS audience better engage.

Comments for the author

MAJOR POINTS:
1. Quantification of variant stability- this is key to interpreting any of these results. I appreciate they are overexpressed, polyclonal lines but somehow linking across all these 13 samples, across all of these assays, we need to know are they (1) destabilizing and/or (2) disrupting protein interaction

© 2022. Published by The Company of Biologists under the terms of the Creative Commons Attribution License (https://creativecommons.org/licenses/by/4.0/).

interfaces? This would be in Figure 1 before your holocomplex analysis- could authors look at mRNA expression level of GFP tag and normalize to protein GFP form to get a proxy of stability? Not ideal- FlpIN or some other single copy approach would be more finessed but beyond the scope of this study.
2. Schematic of holocomplex- Figure 1C- highlight what you have pulled on for the AP and color code to match your interactors in a schematic. Also, for good form, you should have GFP alone control to show none of these interactors are spurious, particularly at low levels of expression.
3. Dominant negative proteins, cilia phenotypes and various KO models- it features prominently here, and in previous Nakayama work, but the evidence that this truncated protein exists is less clear and at what level. Proteomics summarizing the peptides from parental RPE-1, WDR34 KO (Stephens lab Vuolo et al short cilia) and WDR34 KO (Nakayama lab here, Tsurumi et al MBoC paper- 2/4 clones long cilia, 2/4 short cilia) would help nail this as not being random genome editing allele artifact- can we find these truncated peptides by MS? Also to note here, unless these alleles are heterozygous, they are not dominant-negative (must affect wild type gene product in the same cell- so you'd see an effect heterozygously) but gain-of-function/neomorph? (Line 220 for example)

MINOR POINTS
1. Clinical table- summarize the patient phenotypes in a table perhaps, someway of organizing into severity. The text in this section is not very helpful and makes for dense reading. Tell us just what we need to know to understand the experiments- different clinicians will describe similar phenotypes differently, so don't get bogged down in detail.
2. Line 215: 'more complete knockout' this absolutely needs quantification unbiasedly by MS- show peptide and levels.
3. Line 535: How many cell lines did you look at- one polyclonal cell line per construct. Add in details.
4. Line 548: Add in the fixations used specifically for each antibody listed- this is very helpful for your readers.

TEXTUAL EDITS
1. Line 40: 'skeletal deformation'- skeletal dysplasias?
2. Line 43: Jeune is also known as asphyxiating throracic dysplasia (non-lethal) whereas lethal short-rib polydactyly. Syndromes are also not italicized unless they use someone's name (Jeune's but not short-rib polydactyly).
3. Throughout: human genes all caps and italics (i.e. investigated WDR34 mutations) and throughout your intro DYNC2H1 etc 4. ABSTRACT: needs a hook. Why is this important/what is the question.
5. INTRODUCTION: confusing list of clinical descriptions. Simplify and highlight why this is important.
6. Line 122: 'hypotrophic lungs' correct not hyper.
7. Throughout: protein changes for human nomenclature should be p.T354M, like your c.1061C>T.
8. Line 132: Add a reference for digenic ciliopathy inheritance likely Davis et al. Nat Genet. 2011 Mar; 43(3): 189–196.
9. Line 137: Schmidts cell work was all knock-down- 'Knockdown and knock out models of WDR34….).
10. Line 149/150: 'Indeed, this may also be a major impact of the clinical WDR34 Q158* mutation (Huber et al., 2013).' This seems like it belongs in the discussion…. More so as the mutation lies within the WD repeats so chances of structure instability are much stronger than those in the less structured N-terminal region.
11. Line 186: 'were similar to'?
12. Throughout text figures: proteins including ARL13B should be all caps, same for Smoothened (SMO).
13. Line 310-311: 'Mutations in different regions of WDR34 can lead to a common phenotype, whereas those in close proximity can lead to a different set of cilia defects.' This doesn't make sense to me. Like back to stability (See major point), this you do not address at all.
14. Line 448: both fibroblasts and epithelial cells like your RPE-1 ciliate via the intracellular pathway, do you mean polarized epithelial like MDCKs which dock?
15. Line 479/480: ' Cilia function is both cell type and tissue specific'- yes and no your mutations affect one of the core cilia machineries implicated in retrograde trafficking in all cell types, they should all be similarly affected, although accumulated cargos and adaptors would be predicted to be different.

© 2022. Published by The Company of Biologists under the terms of the Creative Commons Attribution License (https://creativecommons.org/licenses/by/4.0/).

16. Line 556: Missing bracket around Santa Cruz. Triton should be capitalized Line 561 and which version spelled out.
17. Line 587: Specifiy how many samples were run for proteomics- experimental replicates, technical triplicates etc.
18. Missing statistics: Which tests were run etc, ideally this should be in the figure legend but at a minimum in the methods.

First revision

Author response to reviewers' comments

We thank both reviewers for the very helpful and insightful comments. We have sought to address them all through extensive changes to the text and figures along with the inclusion of new data.
We hope that this will now prove acceptable for publication in JCS.

Reviewer 1 Comments for the Author:
Main concern:

The majority of WDR34 mutants appear to be expressed at significantly lower levels than the WT control, which complicates the interpretation of the phenotypes. In cases where the mutant phenotype is different from WT, it is unclear to what extent this is due to differences in expression levels. Differences in expression levels among the mutants is another confounding factor. For example, when discussing the effects of R447W and R447Q (lines 382 - 388), the authors state that “the differences between the Q and W at this position suggests some critical differences”. Judging by the immunoblot in Figure 1B, these “critical differences” may well come down to different expression levels. The authors should acknowledge the potential impact of variable protein levels.

The authors should also provide an explanation for this variability in expression levels. Do some mutations prevent proper folding of WDR34, resulting in degradation of the mutant protein? One simple way to test this would be to inhibit proteasome activity, which in case of a destabilizing mutation would be predicted to increase mutant protein levels.

This is a good point that goes along with those made about stability which we address elsewhere. We have added a specific sentence relating to p.R447W/Q in the results section to acknowledge this and deal with this further in relation to the data in new Figure 2. These data show some variability in stability which we describe in the text. As with other data sets there is no direct correlation between stability and phenotypes, but these data do provide some insight into related mutants e.g. R447W and p.R447Q as we describe in the text (line 444). We also retain the original comparison of expression levels in the Supplementary material.

The authors state that FACS was used in the generation of the cell lines but don’t mention single-cell sorting: are these cell lines clonal? If so, were expression levels similar among different clones, i.e. was it impossible to obtain mutant expression levels that are similar to the WT control?

Cells were sorted but not at the single cell level. We retained the use of pools by choice to avoid issues of clonality. This has been clarified in the text.

Specific comments:

- Title: is “clinical mutations” a commonly used term? Maybe change to “disease-causing mutations” or "disease-associated mutations".

We have amended this throughout - thank you for suggesting a much better phrasing.

© 2022. Published by The Company of Biologists under the terms of the Creative Commons Attribution License (https://creativecommons.org/licenses/by/4.0/).

- Line 41: add “respectively”.

Added

- Lines 45/46: “different spectrums of phenotypes” and “distinct profile of phenotypes” is repetitive.

Amended.

- Figure 1B: the immunoblot needs to be of better quality so that relative expression levels can be properly assessed. The GAPDH signal is overexposed, which makes it difficult to judge loading. The GFP signal for WT GFP-WDR34 is also overexposed. The “RPE-1”, “34KO+GFP”, and “WT” samples should be included in both blots, not just in the one on the left.

This blot has been moved to the Supplement (the two gels are from the same experiment; we delineate separate gels clearly as is good practice) and replaced with the stability experiment in Figure 2B. We did not replace this to include controls on both blots as we have very limited capacity with the first three listed authors all having left the lab.

- Figure 1C: A description of the proteomic analysis should be included in the materials and methods section. It would also be good to confirm at least some of these differences by immunoblot (for example DYNC2H1 levels in samples R206C vs T354M), but this is only a suggestion.

We have added fuller description to methods, antibody availability makes validation challenging and is not something previously required to support our quantitative proteomics approaches.

- Line 193: “Interestingly, apart from WDR34-K436R, interaction with DYNLRB1 was reduced by more than half in all WDR34 point mutants located in a WD domain, suggesting defective assembly of DYNLRB1 in dynein-2 may be a common phenotype in WDR34-related disorders”.

The relative abundance of DYNLRB1 in the control sample “WT-WDR34” is also reduced by more than half, so I don’t understand how the authors came to this conclusion.

We apologise sincerely for two mistakes here: this column on the plot was in fact from cells expressing GFP only. We have now removed this as the normalization to WDR34 is not appropriate as these cells do not express GFP-WDR34 only GFP. Instead, we now include an additional plot (Fig. 2D) showing the abundance of dynein-2 subunits when analysed relative to GFP only i.e. confirming the specificity and efficacy of our pull-downs. We have modified the text and Figure legend accordingly. We also now explain Figure S4 within the text but retain this as a Supplementary file for clarity.

-

- Figure 2A: why is the GFP signal in “34KO+GFP” so spotty?

This is a result of chemical fixation. The image has now been replaced with another from the same data set (New Fig. 2).

- Lines 238 - 240: repetition

Deleted.

- Figure 4: Given the non-specific nuclear dots of the IFT140 staining, it would be helpful to add an arrow to the IFT140 images that points at the ciliary base.

Arrows added.

Line 250/Figure 4: “This complicates analysis and so we have not quantified these data but instead scored for the robust presence of IFT140 by eye.” Do the authors mean that the unspecific signal prevents *automated* image analysis?

© 2022. Published by The Company of Biologists under the terms of the Creative Commons Attribution License (https://creativecommons.org/licenses/by/4.0/).

Scoring localization "by eye" is also a form of quantification, is it not?

Agreed – we have added "we have not applied automated quantification to these data"

- Line 259: "We next investigated the localization of the IFT-B protein, IFT88, as a well-characterized reporter of IFT defects." Why a reporter of IFT "defects"?

Now clarified

- Line 265: "WDR34 WT rescued IFT88 distribution from the cilium length." Why cilium "length"?

Deleted

- Line 308: why "unfortunately"?

In the sense we had hoped for some structure function correlation – now deleted.

- Line 354: "Modelling of the mutations indicated that A341V leads to instability of the beta-propellor structure, with the mutant valine clashing with the first strand of the next propellor blade.
Instead, T354 may form important hydrogen bonds important for maintaining structure stability, which are lost upon mutation into methionine." How was this modeling performed?

On reflection, we have now removed this as we do not have the capacity to undertake this for all mutants.

- Figure S3: Include the WT sample.

The WT sample is already included in these schematics as the circle. The figure legend states "In each case, the circle indicates a ratio of 1 between mutant and wild-type WDR34 (i.e. no impact)".

- Figure S4: It would be useful to move this summary table to the last main figure. Rather than just "yes" or "no", the extent of the effect could also be indicated (e.g. "+" vs "++").

We have modified this table and updated it to include as new Figure 8.

Reviewer 2 Advance Summary and Potential Significance to Field:
Ciliopathies are an expanding group of genetics disorders characterized by defects in the structure and/or function of cilia, small microtubule-based projections found on most mammalian cell types. There is a growing disconnect between genotype-phenotype correlations between ciliopathy patients in terms of expressivity and penetrance of clinical phenotypes even between individuals with mutations in the same gene, sometimes even same mutation. The reason for this pleiotropy is not well-understood but impinges on our clinical diagnosis and management of ciliopathy patients. Better ways of linking genotype to molecular and cellular phenotypes to inform on physiology are needed. Shak et al. attempt to do this here for the variants of the IFT dynein-2 intermediate chains DYNC2I2/WDR34 implicated in a spectrum of human skeletal dysplasias. They use a KO human ciliated epithelial line to over-express 13 disease-causing variants to look at molecular motor assembly by proteomics, as well as cilia structure (IFT trafficking by IF) and function (SMO localization by SAG stimulation). The narrative needs attention in terms of what this study is trying to do upfront and why it should target an audience like JCS as opposed to Human Molecular Genetics, for example, where these types of studies normally sit. Additional figure panels to help the audience understand the molecular asymmetry of dynein-2 with a schematic, where colors match your IP legends in figure 1C for example, possibly highlighting the subcomplexes you see partially assembling.

We now include a colour-coded schematic as Figure 2C

It would also benefit from a systemic structural analysis of the mutations (from three papers) through a single predictive pipeline in terms of pathogenicity- (at a minimum PolyPhen, SIFT and

© 2022. Published by The Company of Biologists under the terms of the Creative Commons Attribution License (https://creativecommons.org/licenses/by/4.0/).

Grantham scores, but there are fancier tools out there too…)- a table such that it is a ranked list of predicted severity, is this what you see in your cell assays? Finally, a model summarizing what you have found at the end would help bring together all these experimental strands which currently feel very frayed together again, what is the punchline of these analyses.

This is now included as Supplementary Table 2 and referred to in relevant places in the text. A summary of our data is in Table form as Supplementary Figure 5 (this is an amended version of the previous Table showing more detail as requested by the other reviewer).

This is important and timely work but some additional efforts to increase accessibility of data and messaging would help a JCS audience better engage.

Thank you for your positive evaluation and extremely helpful comments. We have reworked some of the text to better define the objectives fo the study. In terms of our choice of journal, our work does not include the identification of any new mutations in WDR34 and therefore we did not think this suitable for a journal such as Human Molecular Genetics. Indeed, JCS has published extensively on dynein-2 and cilia biology; furthermore, we noted the upcoming special issue relating to Motors in Cell Biology and considered our extensive cell biological analyses to be highly suitable making this our first choice for submission.

Reviewer 2 Comments for the Author:
MAJOR POINTS:

1. Quantification of variant stability- this is key to interpreting any of these results. I appreciate they are overexpressed, polyclonal lines but somehow linking across all these 13 samples, across all of these assays, we need to know are they (1) destabilizing and/or (2) disrupting protein interaction interfaces? This would be in Figure 1 before your holocomplex analysis- could authors look at mRNA expression level of GFP tag and normalize to protein GFP form to get a proxy of stability?

Cell lines are indeed polyclonal. We now include data relating to stability in figure 2 as suggested by the other reviewer. We consider this the best assessment of stability that we can make. We also clarify explanations of our proteomics data which we consider addresses protein interactions.

2. Schematic of holocomplex- Figure 1C- highlight what you have pulled on for the AP and color code to match your interactors in a schematic. Also, for good form, you should have GFP alone control to show none of these interactors are spurious, particularly at low levels of expression.

This is now included as new Figure 1C

3. Dominant negative proteins, cilia phenotypes and various KO models- it features prominently here, and in previous Nakayama work, but the evidence that this truncated protein exists is less clear and at what level. Proteomics summarizing the peptides from parental RPE-1, WDR34 KO (Stephens lab Vuolo et al short cilia) and WDR34 KO (Nakayama lab here, Tsurumi et al MBoC paper- 2/4 clones long cilia, 2/4 short cilia) would help nail this as not being random genome editing allele artifact- can we find these truncated peptides by MS? Also to note here, unless these alleles are heterozygous, they are not dominant-negative (must affect wild type gene product in the same cell- so you'd see an effect heterozygously) but gain-of-function/neomorph? (Line 220 for example)

These apparently contradictory data are of course key to understanding the role of WDR34. We had undertaken this work as part of another project but now include this here as a new Figure 1. We consider that this provides definitive outcomes and shows clearly that the N-terminal fragments in CRISPR knockouts and more importantly here, in the p.Q158* mutant, can indeed have negative impacts on function through direct binding to dynein light chains. This supports the conclusions of Tsurumi et al and provides a clear rationale for using their cell line 1-5 for our study. Including this as new Figure 1 sets the scene for the subsequent figures. This work was done with Aakash Mukhopadhyay and Anthony Roberts for another project but is now included here with them as co-authors.

© 2022. Published by The Company of Biologists under the terms of the Creative Commons Attribution License (https://creativecommons.org/licenses/by/4.0/).

MINOR POINTS
1. Clinical table- summarize the patient phenotypes in a table perhaps, someway of organizing into severity. The text in this section is not very helpful and makes for dense reading. Tell us just what we need to know to understand the experiments- different clinicians will describe similar phenotypes differently, so don't get bogged down in detail.

We now include the key information as a Supplementary Table. We ourselves find such descriptions helpful, especially when contained in one place. We have rationalised descriptions within this Supplementary Table and consider that this highlights the clinical complexity of diagnosis and description yet aids readers of JCS to understand the context of the work.

2. Line 215: 'more complete knockout' this absolutely needs quantification unbiasedly by MS- show peptide and levels. Is the reviewer referring to "more complete knockout" in Laura's WDR34KO compared to the Tsurumi KO used to make the WDR34 mutant cells? The introduction gives the information that the residual WDR34 expressed is >110 residues vs 24 residues, but this is not repeated in the results or methods which may understandably cause conclusion. I have now included that the WDR34KO cells used in this study have a p.Val24Alafs*74 mutation in both the results and methods. I have also added "Other WDR34 KO cell lines generated with p.Val119Alafs*4 or p.Thr151Thrfs*41 mutations and encoding similar N-terminal regions also assemble short cilia (Tsurumi et al., 2019)" to further support that it is more larger WDR34 truncations that result in short cilia.

We have added a new section and new Figure 1 that addresses this concern in considerable depth (see also your point 2 above). This work was done with Aakash Mukhopadhyay and Anthony Roberts for another project but is now included here with them as co-authors. We do not restate the findings here, but we consider that these new data reconcile previous differences between cell lines (adding to the value of this work) as well as defining a likely cause for cilia defects arising from the p.Q158* mutation. We have included this as the start of the results section as we consider that it establishes the basis for the cell line used.

3. Line 535: How many cell lines did you look at- one polyclonal cell line per construct. Add in details.

This is now included in the methods: "Pools were selected that expressed the lowest detectable levels of GFP as verified by fluorescence microscopy. We analysed pools to avoid any differences arising from growing our individual clones."

4. Line 548: Add in the fixations used specifically for each antibody listed- this is very helpful for your readers.

Information now added

TEXTUAL EDITS

1. Line 40: 'skeletal deformation'- skeletal dysplasias?

Amended

2. Line 43: Jeune is also known as asphyxiating throracic dysplasia (non-lethal) whereas lethal short- rib polydactyly. Syndromes are also not italicized unless they use someone's name (Jeune's but not short-rib polydactyly).

Amended

3. Throughout: human genes all caps and italics (i.e. investigated WDR34 mutations) and throughout your intro DYNC2H1 etc In most cases we are erring to proteins hence no italics.

We have amended where we are referring to the gene.

© 2022. Published by The Company of Biologists under the terms of the Creative Commons Attribution License (https://creativecommons.org/licenses/by/4.0/).

4. ABSTRACT: needs a hook. Why is this important/what is the question.

We have amended the abstract but remain within the length restrictions.

5. INTRODUCTION: confusing list of clinical descriptions. Simplify and highlight why this is important.

See new Table 1.

6. Line 122: 'hypotrophic lungs' correct not hyper.

Amended

7. Throughout: protein changes for human nomenclature should be p.T354M, like your c.1061C>T.

Amended

8. Line 132: Add a reference for digenic ciliopathy inheritance likely Davis et al. Nat Genet. 2011 Mar; 43(3): 189–196.

Added in context

9. Line 137: Schmidts cell work was all knock-down- 'Knockdown and knock out models of WDR34….).

Amended

10. Line 149/150: 'Indeed, this may also be a major impact of the clinical WDR34 Q158* mutation (Huber et al., 2013).' This seems like it belongs in the discussion…. More so as the mutation lies within the WD repeats so chances of structure instability are much stronger than those in the less structured N-terminal region.

We now clarify his point in relation to new Figure 1.

11. Line 186: 'were similar to'?

Amended

12. Throughout text figures: proteins including ARL13B should be all caps, same for Smoothened (SMO).

Amended

13. Line 310-311: 'Mutations in different regions of WDR34 can lead to a common phenotype, whereas those in close proximity can lead to a different set of cilia defects.' This doesn't make sense to me. Like back to stability (See major point), this you do not address at all.

We have amended this section to clarify our point and relate this to the new data on stability.

14. Line 448: both fibroblasts and epithelial cells like your RPE-1 ciliate via the intracellular pathway, do you mean polarized epithelial like MDCKs which dock?

We have removed this and the Mazo reference – apologies, we were speculating more widely about differences in model systems.

15. Line 479/480: ' Cilia function is both cell type and tissue specific'- yes and no your mutations affect one of the core cilia machineries implicated in retrograde trafficking in all cell types, they should all be similarly affected, although accumulated cargos and adaptors would be predicted to be different.

© 2022. Published by The Company of Biologists under the terms of the Creative Commons Attribution License (https://creativecommons.org/licenses/by/4.0/).

We have added a line to address this.

16. Line 556: Missing bracket around Santa Cruz. Triton should be capitalized Line 561 and which version spelled out.

Amended

17. Line 587: Specifiy how many samples were run for proteomics- experimental replicates, technical triplicates etc.

Amended – we have now expanded this section as requested by the other reviewer and included specific mention of the three independent repeat experiments, we included in the original data available to reviewers and now all proteomics data will be freely available to all linked to the doi should this work be accepted.

18. Missing statistics: Which tests were run etc, ideally this should be in the figure legend but at a minimum in the methods.

Apologies – we included this in most of the figure legends in the initial submission. We have now checked that all are included. No statistical tests were done in Figure 4. We have added the number of cells analysed for Figure 6. For Figure 5 and have included a statistics test and modified the figure file. We also now include statistics for both -/+ SAG conditions in Figure 7 with associated changes in the text and summary in new Figure 8.

Second decision letter

MS ID#: JOCES/2022/260073

MS TITLE: Disease-associated mutations in WDR34 lead to diverse impacts on the assembly and function of dynein-2

AUTHORS: Caroline Shak, Borhan Uddin, Laura Vuolo, Yohei Katoh, Tom Brown, Aakash G Mukhopadhyay, Kate Heesom, Nicola Stevenson, Anthony John Roberts, Kazuhisa Nakayama, and David Stephens
ARTICLE TYPE: Research Article

We have now reached a decision on the above manuscript.

To see the reviewers' reports and a copy of this decision letter, please go to: https://submit-jcs.biologists.org and click on the 'Manuscripts with Decisions' queue in the Author Area. (Corresponding author only has access to reviews.)

As you will see, the reviewers recommended acceptance of the manuscript but made some specific suggestions that will require minor amendments to your manuscript. I hope that you will be able to carry these out so that I can accept your paper.

Please ensure that you clearly highlight all changes made in the revised manuscript. Please avoid using 'Tracked changes' in Word files as these are lost in PDF conversion.

I should be grateful if you would also provide a point-by-point response detailing how you have dealt with the points raised by the reviewers in the 'Response to Reviewers' box. Please attend to all of the reviewers' comments. If you do not agree with any of their criticisms or suggestions please explain clearly why this is so.

© 2022. Published by The Company of Biologists under the terms of the Creative Commons Attribution License (https://creativecommons.org/licenses/by/4.0/).

Reviewer 1

Advance summary and potential significance to field

N/A

Comments for the author

The authors made a great effort to improve the manuscript. All my concerns are addressed in the revised version. The new Figure 1 is a particularly valuable addition.
A couple of minor suggestions:
- In Figure 2A, it would look better if the color of WDR34 was at least somewhat similar to that of WDR34 in the dynein cartoon of Figure 2B (i.e. a shade of green)
- If I understand correctly, the dashed lines below the sequence in Figure S1A represent the sequencing results in the ko mutant, i.e. there is no sequence since this region is deleted. It would be easier to understand if the sequences flanking the deletion were also shown, which would prove that the sequencing actually worked.

Reviewer 2

Advance summary and potential significance to field

Ciliopathies are an expanding group of genetics disorders characterized by defects in the structure and/or function of cilia, small microtubule-based projections found on most mammalian cell types. There is a growing disconnect between genotype-phenotype correlations between ciliopathy patients in terms of expressivity and penetrance of clinical phenotypes even between individuals with mutations in the same gene, sometimes even same mutation. The reason for this pleiotropy is not well-understood but impinges on our clinical diagnosis and management of ciliopathy patients. Better ways of linking genotype to molecular and cellular phenotypes to inform on physiology are needed. The revised manuscript by Shak et al has introduced extensive new experimental data to 'resolve' controvesies with different cell based models of WDR34, in order to unify the work they present here to understand the differential impact of patient identified disease-causing WDR34 variants on the structure and function of the dynein-2 holocomplex in cilia. To me, the real strength of this paper is to look with both wet and dry lab analyses on the effect of disease-causing variants on expression, stability of the holocomplex as well as functional readouts on cilia biology (etc. rates, lengths, trafficking etc). Even with the caveats of overexpression, it makes a very holostic study of structure-function cataloguing of WDR34 disease-causing variants to unpick these complex and enigmatic molecular motors in health and disease.

Comments for the author

I would recommend publication of the study upon very minor text and figure edits to improve accessibility of their paper.
1. Throughout- where you are talking about patients with mutations, the more politically correct term should be used 'patients with disease-causing variants'. Mutation on its own is fine to use, as long as you don't refer to people. I flagged: Line 103, Line 113, Line 552
2. Take abbreviations out of the abstract, put TZ at the first use in text.
3. Line 117- a little confusing to say KO with a truncating protein produced- KO usually reserved for null allele, consider 'KO' or deletion allele where they produce truncated protein products. I think this would help clarify. Again Line 124. Line 132 add in 'validated null' background?
4. Capitalize Smoothened 312 and 313.
5. Add in 337 'large number of isogenic cells' (you avoid the problem of pesky outbreeding in human populations)
6. Awkward Line 360 Consider 'Compound heterozygous WDR34 variants p.Q158* and p.K436R were previously reported in a patient with Jeune syndrome. That this patient presented with Jeune not the more severe SRPS phenotype can be explained by our observations that WDR34 p.Q158* does not disrupt cilium formation on a background where residual WDR34 function exists'
7. Line 400: typo DYNC2H1?
8. Line 452: typo DYNLRB1?

© 2022. Published by The Company of Biologists under the terms of the Creative Commons Attribution License (https://creativecommons.org/licenses/by/4.0/).

9. Line 456: Consider adding 'Whilst, in this study, none of the mutations led to an increased baseline of SMO in cilia, other elements of SHH signaling were not assayed including GLI trafficking and processing, or target gene induction'. Take out the cell type differences.
10. Figure legend 1- all caps for proteins ARL13B in the legend (Line 693 and 702) and in the figure (also AcTUB). To be consistent throughout label the graphs only with one letter aa code (versus three currently). It would be great for your graphs to plot the means of your three experiments instead of the bar graphs (superplots even better).
11. Figure 2 add in protein ladder- mol wt sizes on C
12. Figure 6- how can you tell tip or base from ARL13B? Maybe an arrowhead to highlight base would be helpful here and make sure you cilia are in a similar orientation (if they are not- I am genuinely not sure)
13. Figure S1. Change from three to one letter aa code on the labels of C. All caps ARL13B.
14. Table S1- use the term 'Compound heterozygous with' throughout and title change to 'disease-causing variants' as you talk about people.

Second revision

Author response to reviewers' comments

Reviewer 1 Advance Summary and Potential Significance to Field:

N/A

Reviewer 1 Comments for the Author:
The authors made a great effort to improve the manuscript. All my concerns are addressed in the revised version. The new Figure 1 is a particularly valuable addition.

A couple of minor suggestions:

- In Figure 2A, it would look better if the color of WDR34 was at least somewhat similar to that of WDR34 in the dynein cartoon of Figure 2B (i.e. a shade of green)

- If I understand correctly, the dashed lines below the sequence in Figure S1A represent the sequencing results in the ko mutant, i.e. there is no sequence since this region is deleted. It would be easier to understand if the sequences flanking the deletion were also shown, which would prove that the sequencing actually worked.

Both Figures are now amended.

Reviewer 2 Advance Summary and Potential Significance to Field:
Ciliopathies are an expanding group of genetics disorders characterized by defects in the structure and/or function of cilia, small microtubule-based projections found on most mammalian cell types. There is a growing disconnect between genotype-phenotype correlations between ciliopathy patients in terms of expressivity and penetrance of clinical phenotypes even between individuals with mutations in the same gene, sometimes even same mutation. The reason for this pleiotropy is not well-understood but impinges on our clinical diagnosis and management of ciliopathy patients. Better ways of linking genotype to molecular and cellular phenotypes to inform on physiology are needed. The revised manuscript by Shak et al has introduced extensive new experimental data to 'resolve' controvesies with different cell based models of WDR34, in order to unify the work they present here to understand the differential impact of patient identified disease-causing WDR34 variants on the structure and function of the dynein-2 holocomplex in cilia. To me, the real strength of this paper is to look with both wet and dry lab analyses on the effect of disease-causing variants on expression, stability of the holocomplex as well as functional readouts on cilia biology (etc. rates, lengths, trafficking etc). Even with the caveats of overexpression, it makes a very holostic study of structure-function cataloguing of WDR34 disease-causing variants to unpick these complex and enigmatic molecular motors in health and disease.

© 2022. Published by The Company of Biologists under the terms of the Creative Commons Attribution License (https://creativecommons.org/licenses/by/4.0/).

Reviewer 2 Comments for the Author:
I would recommend publication of the study upon very minor text and figure edits to improve accessibility of their paper.

1. Throughout- where you are talking about patients with mutations, the more politically correct term should be used 'patients with disease-causing variants'. Mutation on its own is fine to use, as long as you don't refer to people. I flagged: Line 103, Line 113, Line 552

Amended throughout and highlighted

2. Take abbreviations out of the abstract, put TZ at the first use in text.

Amended

3. Line 117- a little confusing to say KO with a truncating protein produced- KO usually reserved for null allele, consider 'KO' or deletion allele where they produce truncated protein products. I think this would help clarify. Again Line 124. Line 132 add in 'validated null' background?

Amended

4. Capitalize Smoothened 312 and 313.

Amended

5. Add in 337 'large number of isogenic cells' (you avoid the problem of pesky outbreeding in human populations)

Amended

6. Awkward Line 360 Consider 'Compound heterozygous WDR34 variants p.Q158* and p.K436R were previously reported in a patient with Jeune syndrome. That this patient presented with Jeune not the more severe SRPS phenotype can be explained by our observations that WDR34 p.Q158* does not disrupt cilium formation on a background where residual WDR34 function exists'

Amended

7. Line 400: typo DYNC2H1?

Amended

8. Line 452: typo DYNLRB1?

DYNLRB here is used to indicated both dynein roadblock light chains.

9. Line 456: Consider adding 'Whilst, in this study, none of the mutations led to an increased baseline of SMO in cilia, other elements of SHH signaling were not assayed including GLI trafficking and processing, or target gene induction'. Take out the cell type differences.

Included but at line 550 (which we think was the intended place).

10. Figure legend 1- all caps for proteins ARL13B in the legend (Line 693 and 702) and in the figure (also AcTUB). To be consistent throughout label the graphs only with one letter aa code (versus three currently).

Figures and text amended.

It would be great for your graphs to plot the means of your three experiments instead of the bar graphs (superplots even better).

Now included.

© 2022. Published by The Company of Biologists under the terms of the Creative Commons Attribution License (https://creativecommons.org/licenses/by/4.0/).

11. Figure 2 add in protein ladder- mol wt sizes on C

Now included.

12. Figure 6- how can you tell tip or base from ARL13B? Maybe an arrowhead to highlight base would be helpful here and make sure you cilia are in a similar orientation (if they are not- I am genuinely not sure)

We now include an asterisk to mark the base. This is based on the predominant accumulation of markers such as ARL13B within the base as well as extensive experience of analysis of cilia and regular colabelling with centrin-2 (not included in this study).

13. Figure S1. Change from three to one letter aa code on the labels of C. All caps ARL13B.

Now included. We have also changed Gln158 to Q158 in line with other comments.

14. Table S1- use the term 'Compound heterozygous with' throughout and title change to 'disease-causing variants' as you talk about people.

Text amended along with amending “mutation” to “variant” in line with other comments

---

Third decision letter

MS ID#: JOCES/2022/260073

MS TITLE: Disease-associated mutations in WDR34 lead to diverse impacts on the assembly and function of dynein-2

AUTHORS: Caroline Shak, Borhan Uddin, Laura Vuolo, Yohei Katoh, Tom Brown, Aakash G Mukhopadhyay, Kate Heesom, Nicola Stevenson, Anthony John Roberts, Kazuhisa Nakayama, and David J Stephens
ARTICLE TYPE: Research Article

I am happy to tell you that your manuscript has been accepted for publication in Journal of Cell Science, pending standard ethics checks.

© 2022. Published by The Company of Biologists under the terms of the Creative Commons Attribution License (https://creativecommons.org/licenses/by/4.0/).
